# Factors associated with tuberculosis treatment failure in the Central East Health region of Burkina Faso

**DOI:** 10.11604/pamj.2018.30.293.15074

**Published:** 2018-08-28

**Authors:** Adama Diallo, Désiré Lucien Dahourou, Ter Tiero Elias Dah, Souleymane Tassembedo, Romial Sawadogo, Nicolas Meda

**Affiliations:** 1 National Tuberculosis Control Program, Ouagadougou, Burkina Faso; 2 Centre Muraz, Department of Clinical Research, Bobo-Dioulasso, Burkina Faso; 3 Central East Health Regional Direction, Tenkodogo, Burkina Faso; 4 University of Ouagadougou, Department of Public Health, Ouagadougou, Burkina Faso

**Keywords:** Tuberculosis, treatment, failure, Burkina Faso

## Abstract

**Introduction:**

Tuberculosis treatment failure results in increased risk of morbidity, drug resistance, transmission and mortality. There are few data about tuberculosis treatment outcomes in Burkina Faso. The current study investigated the factors associated with tuberculosis treatment failure in the central east health region of Burkina Faso.

**Methods:**

We conducted a case-control study. All cases of pulmonary tuberculosis failing first-line treatment matched to those who were cured (controls) in the Central Eastern Region were sampled from January 2010 to December 2014. Predictors of treatment failure were determined using multiple conditional logistic regression.

**Results:**

A total of 381 patients with positive microscopic pulmonary tuberculosis were included. Of these 76 cases failed first-line treatment while 305 controls were cured. Weight loss between diagnosis and first sputum-smear examination was significantly associated with the tuberculosis treatment failure [aOR: 2.5, 95% CI: 1.3-4.7]. In addition, the delay from between treatment initiation to first sputum-smear examination, and high bacillary load at the first sputum-smear examination were significantly associated with treatment failure (p<0.001).

**Conclusion:**

Strengthening the relationship between health care services and local communities to improve the follow-up of tuberculosis patients, and improving adherence to tuberculosis treatment among tuberculosis patients with weight loss between treatment initiation and 2-month sputum-smear examination could be useful to reduce the risk of unsuccessful outcome.

## Introduction

Worldwide, Tuberculosis (TB) remains a major public health problem. In 2014, the World Health Organization (WHO) estimated that 28% of all TB cases were recorded from Africa although unevenly distributed throughout the region [1]. Tuberculosis prevalence was estimated at 690 per 100 000 population with an incidence of 834 per 100,000 population in South Africa alone (the most affected country). Recently in Nigeria, the most affected West African country, prevalence and incidence of TB were estimated at 330 per 100 000 and 322 per 100 000 population [1]. Worldwide, TB mortality in 2014 was estimated at 16 deaths per 100,000 population [1]. The incidence of tuberculosis in Burkina Faso and its mortality were estimated at 54 per 100 000 population and 12 per 100 000 population respectively [1]. Burkina Faso began introducing over two decades ago strategies to control TB through a national program set up. The National TB Control Program adopted the Directly Observed Treatment Strategy (DOTS) which was replaced in 2006 by the Stop TB Strategy [2]. The main components of this strategy included short-term standardized treatment regimen, controlled anti-tuberculosis drug intake and prevention of anti-tuberculosis drugs resistance are [3]. Despite the implementation of these strategies, tuberculosis treatment failure remains appreciably high. According to the national tuberculosis surveillance, the rate of tuberculosis treatment failure in Burkina Faso, was estimated at 5% and 4% in 2010 and 2014 respectively. TB treatment failures most likely lead to increased morbidity and mortality [4]. To say the least, 20.5% of previously treated TB cases (failure, relapse, resumption of treatment) were estimated to have developed multidrug-resistant TB (MDR-TB) (defined as resistance to at least isoniazid and rifampin) in 2013 [5]. Identifying factors associated with TB treatment failure will augment the smooth implementation of interventions curtailing anti-tuberculosis drug resistance as well as halting the tuberculosis transmission chain. Several studies have been carried out to identify the determinants of the TB treatment failure in Africa [4, 6, 7] however an all-inclusive data of factors associated with TB treatment failure is yet to be documented in Burkina Faso. The purpose of this study was to identify factors associated with bacteriologically confirmed pulmonary TB treatment failure in the central-eastern health region of Burkina Faso.

## Methods

**Setting**: Our study was conducted in the central-eastern health region of Burkina Faso. In 2014, the Central-Eastern health region composed of seven health districts (Tenkodogo, Koupéla, Pouytenga, Bittou, Ouargaye, Garango and Zabré), including 159 primary health-care and 6 secondary health care centres. Its population was estimated at 1,384,663. In that same year, the incidence of tuberculosis and relapse cases were 24 per 100,000 population [8].

**Tuberculosis screening and management**: Patients suffering from cough which lasted over 14 days were suspected of pulmonary tuberculosis. They were subjected to sputum smear examinations at TB diagnosis and treatment health centre (HCT). The Ziehl-Neelsen's technique was used to identify an acid-fast bacillium in the sputum. Patients with 2 positive smears were labeled as new bacteriologically confirmed pulmonary TB patients. These patients were treated with first-line anti-tuberculosis treatment for six months regimen according to the national TB program guidelines (2 months of rifampicin (R), isoniazid (H), pyrazinamide (Z) and ethambutol (E), plus 4 months of rifampicin and isoniazid [2RHZE/4RH]) [2]. After initiation of treatment, a follow-up was organized at the closest health care centre for patients. Daily intake of their anti-tuberculosis drugs was monitored by a health worker during the first two months of treatment. HIV Rapid diagnosis testing was performed on all consenting TB patients. Conversely, HIV-positive patients were actively screened for TB during every medical visit. TB/HIV co-infected patients received cotrimoxazole as prophylaxis against opportunistic infections. These patients are eligible for antiretroviral treatment according to the national HIV program guidelines [9].

**Study design and population**: We conducted a case-control study among bacteriologically confirmed pulmonary TB patients treated in the eastern-central health region of Burkina Faso from January 2010 to December 2014. Cases and controls were matched individually by age and sex. A case was defined as a patient with a new bacteriologically confirmed pulmonary TB aged more than 15 years with TB treatment failure. Treatment failure has been defined as a TB patient whose sputum smear examination is positive at month 5 or later during TB treatment. A control was a patient with a new bacteriologically confirmed pulmonary TB who successfully completed treatment (sputum-smear examination negative at 5-month and 6-month of treatment). To increase the power of our study, we matched each case to 4 controls. The controls were selected randomly in the same sex group and in age group-interval of 5 years.

**Data collection**: The data were extracted from three registries at the regional tuberculosis diagnostic and treatment centres (TB case management registers, TB treatment and follow-up registers and HIV/TB co-infected treatment registries). The demographics and parameters collected sampled from the registry for each patient included: age when the individual was diagnosed with TB, sex, occupation, distance from patient's home to the closest primary health facility, distance from patient's home to the closest TB diagnosis and treatment care facility, type of directly observed treatment (DOT) centre, weight at diagnosis, weight at 2-month sputum-smear examination, results of 2-month and 5-months sputum-smear examination, bacillary load at diagnosis and at 2-month sputum-smear examination, HIV status.

**Statistical analysis**: We described the characteristics of our study population. Categorical variables were compared by Mac-Nemar χ² tests or the Cochran Mantel-Haenzel χ² test. Quantitative variables were compared by the student t test for paired variables. Conditional logistic regression was used to identify factors associated with TB treatment failure. The outcome variable was “TB treatment failure” (case/control). Independent variables associated with TB treatment failure at 30% threshold in the univariate analysis were included in the multivariate analysis model. The final model was obtained by a manual stepwise descending approach. A P-value < 0.05 was considered as significant. Multivariate analysis was adjusted on HIV status to account for its potential confounding effect. All analysis were done with R (version 3.3.1, 2016).

**Ethical approval**: This study was authorized by the Central Eastern Regional Health Director who sent an information letter to all Medical District Officers and to the Director of the Regional referral hospital. The questionnaires did not mention patient's name to assure the anonymity of the patient.

## Results

**Characteristics of the study population**: A total of 381 cases with bacteriologically confirmed pulmonary TB were included. Of these, 76 were cases of TB treatment failure and 305 were controls (cured) (Table 1). The median age of patients was 37 years [Interquartile Range (IQR): 29-50], 62.7% were men while 45.9% were farmers. Of all the sampled population, about 34% were housewives. For the majority of the patients, the directly observed treatment phase took place at a closest primary health care facility (71.7%) (Table 1). This proportion was significantly higher in cases than in controls (73.7% vs 71.1%, p <0.01). More than half of the patients had a weight increase between diagnosis and 2-month sputum-smear examination. This proportion was significantly higher in controls compared to cases (72.4% vs 48.4%, p <0.001). The bacillary density at the first smear control was significantly higher in cases than in controls (p<0.001) (Table 1 (suite)). HIV co-infection was significantly more prevalent in cases compared to controls (11.8% versus 8.9%, p <0.001).

**Table 1:** Socio-demographic, clinical and biological characteristics of the study patients, Central Eastern Region, Burkina Faso, 2010-2014 (N = 381)

Variable	N=381 n(%)	TB treatment failure Cases (N=76) n(%)	Controls (N=305) n(%)	P-value
Age at last diagnosis, years, median [IQR]	37(29-50)	41.5 (30.0-52.0)	37.0 (28.0-50.0)	< 0.001ᶧ
**Age group, years**				<0.001ᶧᶧ
15-20	16 (4.2)	2(2.6)	14(4.6)	
20-30	89(23.4)	14(18.4)	75(24.6)	
30-40	100(26.2)	18(23.7)	82(26.9)	
40-50	63(16.5)	20(26.3)	43(14.1)	
>= 50	113(29.7)	22(28.9)	91(29.8)	
**Sex**				
Male	239(62.7)	50(65.8)	189 (62.0)	<0.001*
Female	142 (37.3)	26 (34.2)	116(38.0)	
**Occupation**				0.416ᶧᶧ
Farmer	175(45.9)	33 (43.4)	142(46.6)	
Trader	49 (17.1)	13 (17.1)	36 (11.8)	
Housewife	131(34.4)	23 (30.3)	108 (35.4)	
Others	26 (6.8)	7 (9.2)	19 (6.2)	
**Distance from health facility to HCT (Km)**				0.99ᶧᶧ
Resident	123(36.9)	23 (35.9)	100 (37.2)	
≤10 km	26(7.8)	6 (9.4)	20 (7.4)	
10-20 km	62(18.6)	12 (18.7)	50 (18.6)	
20-30 km	26(7.8)	4 (6.2)	22 (8.2)	
30-40 km	58 (17.4)	11 (17.2)	47 (17.3)	
>40 km	38 (11.4)	8 (12.5)	30(11.2)	
Missing	48			
**Distance from residence to health facility (Km)**				0.822ᶧᶧ
Residents	296 (81.3)	64(85.3)	282 (80.3)	
≤5km	22(6.0)	3 (4.0)	19 (6.6)	
5 à 10 km	35 (9.6)	6(8.0)	29 (10.0)	
>10 km	11(3.0)	2 (2.7)	9(3.1)	
Missing	17			
**Place of DOT**				0.007*
HCT	108 (28.3)	20 (26.3)	88 (28.9)	
HSPC	273 (71.7)	56 (73.7)	217(71.1)	
**HCT**				0.360 ᶧᶧ
Bittou	28 (7.3)	6 (7.9)	22 (7.2)	
Garango	25(6.6)	5 (6.6)	20 (6.6)	
Koupela	115 (30.2)	19 (25.0)	96 (31.5)	
Ouargaye	47(12.3)	10 (13.2)	37 (12.1)	
Pouytenga	32 (8.4)	9 (11.8)	23 (7.5)	
Tenkodogo	107 (28.1)	18 (23.7)	89 (29.2)	
Zabre	27 (7.1)	9 (11.8)	18 (5.9)	
**Delay of treatment (days)**				0.523 ᶧᶧ
≤ 1	173 (45.4)	30(39.5)	143 (46.9)	
2-7	163 (42.8)	34 (44.7)	129 (42.3)	
7-14	24 (6.3)	7 (9.2)	17 (5.6)	
>14	21 (5.5)	5 (6.6)	16 (5.2)	

* chi-2 de macnemar

ᶧ Student test for matched series

ᶧᶧ chi-2 cochran Manthel hantzel Wilcoxon, IQR =interquartile rang, HCT= diagnosis and treatment center ; HSPC=Health and Social Promotion Center ; DOT= directly observed treatment; Km=kilometer

**Table 1 (suite):** Socio-demographic, clinical and biological characteristics of the study patients, Central Eastern Region, Burkina Faso, 2010-2014 (N = 381)

Variables	N=381 n(%)	Case (N=76) n(%)	Controls (N=305) n(%)	P-value
**Delay of 1^st^ sputum-smear examination (months)**				0.180 **
<= 2 months	74(19.4)	18 (23.7)	56 (18.4)	
2 - 3 months	267(70.1)	46 (60.5)	221(72.5)	
3 - 4 months	26(6.8)	8 (10.5)	18(5.9)	
>4 months	14(3.7)	4 (5.3)	10(3.3)	
**Weight at diagnosis (kgs)**				0.181 **
[20 -40]	68(17.8)	13 (17.1)	55(18.0)	
[40-60]	269(70.6)	50(65.8)	219(71.8)	
>60	44(11.5)	13(17.1)	31(10.2)	
**Weight at 1^st^ sputum-smear examination (Kgs)**				0.383 **
[20 -40]	44 (13.6)	11(17.7)	33 (12.6)	
[40-60]	232 (71.8)	42 (67.7)	190 (72.8)	
>60	47 (14.6)	9( 14.5)	38 (14.6)	
Missing	58			
**Weight variation**				< 0.001 **
increase	219(67.8)	30 (48.4)	189 (72.4)	
decrease	104 (32.2)	32 (51.6)	72 (27.6)	
Missing	58			
**Smear grade category at diagnosis**				0.164 **
PTB smear scanty	21 (5.5)	2 (2.6)	19 (6.2)	
PTB smear 1+	44(11.5)	7 (9.2)	37 (12.1)	
PTB smear 2+	57 (15.0)	8 (10.5)	49 (16.1)	
PTB smear 3+	259 (68.0)	59 (77.6)	200 (65.6)	
**Smear grade category at 1^st^ sputum-smear examination**				< 0.001 **
Negative	241(63.3)	27 (35.5)	214 (70.2)	
PTB smear scanty	38 (10.0)	9 (11.8)	29 (9.5)	
PTB smear 1+	62(16.3)	23 (30.3)	39 (12.8)	
PTB smear 2+	31(8.1)	12 (15.8)	19 (6.2)	
PTB smear 3+	9 (2.4)	5 (6.6)	4 (2.4)	
**HIV status**				< 0.001 *
Negative	339 (91.1)	67(88.2)	272 (91.1)	
Positive	33 (8.9)	9 (11.8)	24 (8.9)	
Missing	9			

* chi-2 de macnemar , ᶧ Student test for matched series

** chi-2 cochran Manthel hantzel Wilcoxon,

IQR =interquartile; PTB= pulmonary tuberculosis

**Univariate analysis**: Four variables were associated with TB treatment failure at the threshold of 30%. These variables were weight at diagnosis, change in patient weight between diagnosis and first sputum-smear examination, time from treatment initiation to first sputum-smear examination and bacillary load at first sputum-smear examination (Table 2).

**Table 2:** Factors associated with tuberculosis treatment failure in the Central-Eastern region, Burkina Faso, 2010-2014. Univariate analysis

Variables	N=381	TB treatment failure Cases (%)	OR	95%CI	P-value
**Occupation**					0.436
Housewife	131	17.6	1.0	-	
Farmer	175	18.9	0.9	0.3-2.6	
Trader	49	26.5	1.5	0.5-4.5	
Others	26	26.9	1.5	0.4-5.5	
**Distance from residence to health facility (Km)**					0.431
Resident	296	21.6	1.0	-	
≤5km	22	13.6	0.6	0.2-2.4	
5 à 10 km	35	17.1	0.8	0.3-2.0	
>10 km	11	18.2	0.9	0.2-4.4	
Missing	17	5.9	0.2	0.0-1.8	
**Distance from health facility to HCT (Km)**					0.939
Resident	123	18.7	1.0	-	
≤10 km	26	23.1	1.4	0.5-4.0	
10-20 km	62	19.4	1.1	0.5-2.5	
20-30 km	26	15.4	0.9	0.3-2.7	
30-40 km	58	19.0	1.1	0.5-2.5	
>40 km	38	21.1	1.2	0.5-3.1	
Missing	48	25.0	1.6	0.7-3.6	
**Place of DOT**					0.589
HCT	108	18.5	1.0	-	
HSPC	273	20.5	1.2	0.7-2.1	
**HCT**					0.384
Tenkodogo	107	16.8	1.0	-	
Garango	25	20.0	1.4	0.5-4.1	
Koupela	115	16.5	1.0	0.5-2.1	
Ouargaye	47	21.3	1.4	0.6-3.3	
Pouytenga	32	28.1	2.3	0.9-6.4	
Bittou	28	21.4	0.7	0.2 -1.2	
Zabre	27	33.3	2.8	1.0-7.9	
**Delay of treatment (days)**					0.544
≤1	173	17.3	1.0	-	
2-7	163	20.9	1.2	0.7-2.2	
7-14	24	29.2	2.0	0.7-5.4	
>14	21	23.8	1.5	0.5-4.4	
**Delay of 1st sputum-smear examination (months)**					0.189
≤2 **months**	74	24.3	1.0	-	
2 - 3 **months**	267	17.2	0.6	0.3-1.6	
3 - 4 **months**	26	30.8	1.4	0.5-3.7	
>4 **months**	14	28.6	1.2	0.3-4.4	
**Weight at diagnosis**					0.206
[20-40]	68	19.1	1.0	-	
[40-60]	269	18.6	0.8	0.4-1.7	
>60	44	29.5	1.6	0.6-3.8	
**HIV status**					0.529
Negative	339	19.8	1.0	-	
Positive	33	27.3	1.6	0.70-3.69	
Missing	9	19.9	< 0.001	< 0.01-inf	

OR = odds ratio; CI = confidence interval; HCT= diagnosis and treatment center ;HSPC=Health and Social Promotion Center ; DOT= directly observed treatment

**Multivariate analysis**: after adjusting for HIV co-infection, three variables were significantly associated with TB treatment failure; time from treatment initiation to first sputum-smear examination, change in patient between diagnosis and first bacillary control and bacillary load at first sputum-smear control (Table 3).The weight loss between the diagnosis and the first sputum-smear examination was significantly associated with the TB treatment failure independently to other variables [aOR: 2.5, 95% CI: 1.3-4.7] (Table 3). The bacillary load at first sputum-smear examination was significantly associated with the odds of TB treatment failure (p <0.001), with dose-response effect.

**Table 3:** Factors associated with tuberculosis treatment failure in the Central-Eastern region, Burkina Faso, 2010-2014. Univariate and multivariate analysis

Variables	N=381	Case	OR	CI 95%	p	aOR	95%CI	P-value
**Delay of 1st sputum-smear examination (months)**					0.189			0.039
≤ 2 months	74	24.3	1.0	-		1.0	-	
2-3 months	267	17.2	0.6	0.33-1.63		0.8	0.4-1.7	
3-4 months	26	30.8	1.4	0.52-3.69		2.2	0.7-6.9	
>4 months	14	28.6	1.2	0.35-4.44		4.9	1.1-21.2	
**Weight variation**					0.002			0.005
Increase	219	13.7	1.0			1.0	-	
Decrease	104	30.8	2.8	1.55-5.00		2.5	1.3-4.7	
Missing	58	24.1	2.0	1.01-4.10		2.9	1.3-6.7	
**Smear grade category at 1st sputum-smear examination**					< 0.001			< 0.001
Negative	241	11.2	1.0	-		1.0	-	
PTB smear scanty	38	23.7	5.7	2.77-11.60		2.4	1.0-6.2	
PTB smear 1+	62	37.1	6.8	2.74-16.96		6.1	2.8-13.2	
PTB smear 2+	31	38.7	12.8	3.06-53.74		8.3	3.0-22.7	
PTB smear 3+	9	55.6	2.4	1.01-5.54		17.6	3.6-85.0	
**HIV status**					0.529			0.809
Negative	339	19.8	1.0	-		1.0	-	
Positive	33	27.3	1.6	0.70-3.69		1.4	0.5-4.2	
Missing	9	19.9	< 0.001	< 0.01-inf		-	-	

OR = odds ratio; CI = confidence interval; aOR = adjusted odds ratio; PTB= pulmonary tuberculosis

## Discussion

Our study reported that the delay between treatment initiation and first sputum-smear examination as well as weight loss between diagnosis and first sputum-smear examination were factors associated with first line pulmonary tuberculosis treatment failure. Furthermore, high bacillary load at the first sputum-smear examination was also associated with first line pulmonary tuberculosis treatment failure. Weight was monitored at diagnosis and throughout the subsequent visits according to Burkina Faso's TB treatment guidelines. Our results showed that the patient's weight loss between the diagnosis and the first bacilloscopic examination increased the odds of treatment failure. This result was consistent with findings of previous studies conducted in others settings [10-12]. Recent studies showed an association between weight loss in the second month following treatment initiation and multidrug resistant TB [13, 14]. This result further expounds on the importance of using weight as a TB treatment monitoring indicator in resources limited settings where diagnosis tools are not always available in primary health care setting. Our study again confirms the importance of developing nutritional supplementation support for tuberculosis infected patients.

We noticed that sputum-smear positivity at 2-month was a factor associated with treatment failure. Patients with higher bacillary load at this examination were more likely to experience treatment failure [6, 15-18]. The sputum-smear examination is normally performed at the end of the 2^nd^ month of the anti-tuberculosis treatment [2]. The first two months of tuberculosis treatment represent the initial phase of treatment. During this intensive treatment phase, the medication intake is supervised preferably by a health worker. It is during this phase that the smear examination is most often negative [18]. In the context of Burkina Faso, sputum-smear positivity at 2-month could be explained by a non-systematic supervision of anti-tuberculous treatment which leads to low adherence to treatment. It could also be explained by the presence of a primary resistance to anti-tuberculosis drugs leading to a selection of multi-resistant bacilli. Atypical mycobacteria that are not sensitive to the first line regimen of TB drugs [2 (RHZE)/4 (RH)] might also explain this finding. In fact, sputum-smear examination with Ziehl-Neelsen technique can identify acid-fast bacilli (AFB). But, these AFB could be mycobacterium tuberculosis or atypical mycobacteria. Indeed, Zida et al reported a high incidence of atypical mycobacteria (11%) in patients with pulmonary tuberculosis with a positive bacillary microscopy in the Hauts-Bassins region in western Burkina Faso [19]. Furthermore, we have established that TB patients who initiated treatment with high bacillary load at the 2-month sputum-smear examination were more likely to have treatment failure compared to those with lower bacillary load. High bacillary load also might be related to late diagnosis when patients are already at advanced stage in their disease. These findings highlight the importance to design and implement strategies to reduce the delay between TB symptoms onset and diagnosis. Patient should also be screened for atypical bacilli at diagnosis.

Our results showed that the delay between treatment initiation and the first control was associated with TB treatment failure, patients with a delay in 2-month sputum-smear examination being more likely to experience TB treatment failure. Our results highlight follow-up challenges in TB patients included in this program. These difficulties are, on one hand due to the irregularity of the access to the smear-based follow-up examination by tuberculosis patients due to the low capacity of laboratories in the region (scarcity of reagents, laboratory technicians shortage) and on the other hand the poor application of the directly observed treatment. These findings underlined the need to strengthen laboratory capacity and to improve collaboration with community health workers to monitor treatment. Unlike findings reported in some studies [20, 21], ours showed that TB/HIV co-infection was not predictor of TB treatment failure. Our results are consistent with those of other [7, 22]. This could be explained by a high rate of mortality rate prior to the 5th-month sputum-smear examination among TB/HIV co-infected patients, resulting in a low rate of inclusion of these cases in our study. For their HIV infection, TB/HIV co-infected patients were followed up in a HIV treatment referral centre which is not fully integrated with TB treatment centres. Thus, HIV/TB co-infected patients had to travel long distances between TB management centre and antiretroviral therapy centre. These pathways could lead to a lack of sputum-smear examination, resulting to an under-reporting of TB treatment failure cases among the TB/HIV co-infected patients. In addition, in this study, there were lot of missing data on HIV status, which could increase the under-reporting of treatment failure among these patients.

**Strengths and limitations of the study**: Our study has some limitations. Data for treatment outcomes were available only for patients who underwent smear tests. Therefore, cases and controls are probably not representative of all cases of TB patients treated in the entire Central Eastern health region over the study period. Moreover, the retrospective nature of data collection was accompanied with large number of missing data. In addition, confounder variables such as discontinuation of treatment, antiretroviral therapy for TB/HIV co-infected patients, comorbidities (diabetes, hepatitis) could not be collected. These factors may influence adherence and therefore the effect of the TB treatment. Height was not reported and therefore it was impossible to study the association between the Body Mass Index (BMI) and TB treatment failure. Despite these limitations, our study is the first to use programmatic tuberculosis data from an overall health region in Burkina Faso to identify factors associated with TB treatment failure.

## Conclusion

Weight loss from the initiation of TB treatment to the first sputum-smear examination, delay in 2-month sputum-smear examination and the high bacillary load at 2-months smear examination were determinants of first line anti-tuberculosis treatment failure. The Improvement of tuberculosis patient's outcomes in East Health Region of Burkina Faso requires strengthening collaborative strategies with community health workers in TB treatment monitoring. A strategy of systematic research for drugs resistant tuberculosis and non-mycobacterium tuberculosis in any new case of tuberculosis with a decrease in weight between the treatment initiation and the 2-month sputum-smear examination could improve treatment outcomes. For effective monitoring in co-infected patients, integration of tuberculosis and HIV care services is necessary. There is an urgent need to actively monitor cases of TB treatment failure in Burkina Faso in order to achieve the goals of the End-TB strategy by 2035.

### What is known about this topic

- Lack or poor adherence to TB drugs predisposes patients to treatment failure.

### What this study adds

- In the context of Burkina Faso, a weight loss after TB treatment initiation, delay in the 2-mont sputum-smear examination, and a high bacillary load at the 2-month sputum-smear examination were predictors of TB treatment failure. These factors could be identified early and reinforcement of treatment adherence could be implemented to improve TB treatment outcomes. There is an urgent need for the national tuberculosis control program to make available bacilloscopy examination tests according to the requirement of the scheduled protocol.

## Competing interests

The authors declare no competing interests.
